# Evidence for Circulation of the Rift Valley Fever Virus among Livestock in the Union of Comoros

**DOI:** 10.1371/journal.pntd.0003045

**Published:** 2014-07-31

**Authors:** Matthieu Roger, Marina Beral, Séverine Licciardi, Miradje Soulé, Abdourahime Faharoudine, Coralie Foray, Marie-Marie Olive, Marianne Maquart, Abdouroihamane Soulaimane, Ahmed Madi Kassim, Catherine Cêtre-Sossah, Eric Cardinale

**Affiliations:** 1 Centre de Coopération Internationale en Recherche Agronomique pour le Développement (CIRAD), UMR 15 CMAEE, Sainte Clotilde, La Réunion, France; 2 Institut National de la Recherche Agronomique (INRA), UMR 1309 CMAEE, Sainte Clotilde, La Réunion, France; 3 Centre de Recherche et de Veille sur les Maladies Émergentes dans l'Océan Indien (CRVOI), Plateforme de Recherche CYROI, Sainte Clotilde, La Réunion, France; 4 Vice-Présidence en Charge de l'Agriculture, l'Elevage, la Pêche, l'Industrie, l'Energie et l'Artisanat, Mdé, Moroni, Union des Comores; 5 Unité de Virologie, Institut Pasteur de Madagascar, Antananarivo, Madagascar; Centers for Disease Control and Prevention, United States of America

## Abstract

Rift Valley fever virus (RVFV) is an arthropod-borne phlebovirus reported to be circulating in most parts of Africa. Since 2009, RVFV has been suspected of continuously circulating in the Union of Comoros. To estimate the incidence of RVFV antibody acquisition in the Comorian ruminant population, 191 young goats and cattle were selected in six distinct zones and sampled periodically from April 2010 to August 2011. We found an estimated incidence of RVFV antibody acquisition of 17.5% (95% confidence interval (CI): [8.9–26.1]) with a significant difference between islands (8.2% in Grande Comore, 72.3% in Moheli and 5.8% in Anjouan). Simultaneously, a longitudinal entomological survey was conducted and ruminant trade-related information was collected. No RVFV RNA was detected out of the 1,568 blood-sucking caught insects, including three potential vectors of RVFV mosquito species. Our trade survey suggests that there is a continuous flow of live animals from eastern Africa to the Union of Comoros and movements of ruminants between the three Comoro islands. Finally, a cross-sectional study was performed in August 2011 at the end of the follow-up. We found an estimated RVFV antibody prevalence of 19.3% (95% CI: [15.6%–23.0%]). Our findings suggest a complex RVFV epidemiological cycle in the Union of Comoros with probable inter-islands differences in RVFV circulation patterns. Moheli, and potentially Anjouan, appear to be acting as endemic reservoir of infection whereas RVFV persistence in Grande Comore could be correlated with trade in live animals with the eastern coast of Africa. More data are needed to estimate the real impact of the disease on human health and on the national economy.

## Author Summary

Rift Valley fever (RVF) is a viral disease transmitted by mosquitoes to ruminants. The disease may affect humans and has a great impact on the economy of the affected country. RVF occurs mostly in African countries, but epidemics have been reported in Madagascar and in the Arabian Peninsula. In the Union of Comoros, RVF virus (RVFV) has been suspected of continuously circulating since 2009 without any notifications of the typical clinical signs by the Comorian Animal Health Services. From April 2010 to August 2011, we conducted a livestock longitudinal survey in Grande Comore, Moheli and Anjouan. Our study aimed to detect RVFV-specific antibody acquisitions in cattle and goats. Simultaneously, a longitudinal entomological survey was conducted to describe the diversity of mosquitoes in the study zones and ruminant trade-related information was collected. Our investigations showed that Comoros ruminants acquired RVFV-specific antibodies all along the year and particularly in Moheli during the dry season. Our findings suggest a complex RVFV epidemiological cycle in the Union of Comoros with probable inter-islands differences in RVFV circulation patterns. The disease appears to be endemic in Moheli and potentially Anjouan, but the persistence of the disease in Grande Comore could be correlated with trade in live animals with the eastern coast of Africa.

## Introduction

Rift Valley fever (RVF) is an arthropod-borne zoonotic disease caused by a RVF virus (RVFV), a member of the *Phlebovirus* genus of the family Bunyaviridae [1]. RVFV causes significant morbidity and mortality among sheep, goats, cattle and also affects humans. In livestock, abortion storms and high mortality observed among the younger animals cause significant economic losses [2], [3]. Humans are usually infected by contact with infectious animal tissues through inhalation or aerosols generated by slaughtering and necropsy [4]. Arthropod vectors play an important role during the onset of epidemic and inter-epidemic periods [5]. In endemic areas, RVFV is maintained in the environment through an enzootic vertebrate-arthropod cycle [6]. RVFV has been isolated from many vectors in the field [7], such as ticks and sand flies which are able to transmit the virus in experimental conditions [8], [9]. However, mosquitoes are the main insects involved in the spread of RVFV during epidemics. RVFV has been isolated from at least 40 species of mosquitoes belonging to 8 genera but only some of them are susceptible and able to transmit RVFV under laboratory conditions [10]. RVF is widely present in Africa and has been spreading to Madagascar and the Arabian Peninsula [11], [12]. In 2007, RVF outbreaks were reported in several eastern and southern African countries [13]. A few weeks later, and for the first time, RVFV was detected in the Comoros archipelago following the hospitalization of a young Grande Comorian boy showing symptoms of severe encephalitis [14]. In addition, during the 2008 and 2009 rainy seasons, outbreaks due to RVFV strains imported from mainland Africa were reported in Madagascar causing 59 confirmed human cases and seven deaths [11], [15].

In Mayotte, the French overseas territory that belongs to the Comoros archipelago, a retrospective study conducted in 2008 confirmed the presence of the disease with 10 human cases infected with RVFV strains genetically closely linked to the 2006–2007 Kenyan isolates [16]. It was also found that the Mayotte livestock has been infected by RVFV prior to 2004 [17]. Regarding the Union of Comoros, in 2011, Roger et al. reported widespread exposure of Comorian livestock with 32.8% of animals shown to be RVFV-seropositive without any notifications of massive abortions or abnormal mortality in the younger animals by the Comorian Animal Health Services. However, the origin of this infection remains unknown [18].

The Union of Comoros is located in the South West Indian Ocean at the northern end of the Mozambique Channel and is considered to be a gateway to islands in the Indian Ocean for various infectious agents imported from mainland Africa. Since 2002, live ruminants are imported from Tanzania and have entered the country without a period of quarantine or a clinical examination [18]. Finally, in the past, animal trade has already affected the country health status on several occasions, with regard to many diseases, like blackleg in 1970 and 1995, the contagious ecthyma in 1999, and the East Coast fever in 2003 and 2004 [19]–[21].

Some of the Culicidae species described in the Comoros archipelago [22] have already been shown to be involved in RVFV transmission. The establishment of RVFV in the Union of Comoros remains unconfirmed and the threat to the Comorian population and neighboring countries needs to be considered.

The trade and resulting movements of ruminants, the composition and abundance of the vector population and many other environmental and anthropological factors determine the nature of the RVF viral cycle. In order to elucidate how RVFV persists in the Union of Comoros, longitudinal and cross-sectional livestock surveys were conducted between April 2010 and August 2011 in six separate geographical zones. Mosquito populations were categorized in parallel over the same period via a longitudinal entomological survey. Additionally trade frequencies were analyzed, providing an estimate of regional ruminant flux and allowing for evaluation of the risk associated with animal importation, and the likelihood of RVFV persistence in the Comoros islands.

## Materials and Methods

### Ethics statement

The research protocol was implemented with the approval of the Vice-Presidency of Agriculture, Fisheries and Environment of the Union of Comoros. No endangered or protected species were involved in the survey. Farmers in each zone gave their verbal consent to be included in the study. Permissions for the blood sample collection were obtained. The animals were bled without suffering. Regarding the trade survey, no personal data were collected, and only information concerning the number of animals travelling from one island to another was taken into account.

### Study zones

The Comoros islands form an archipelago of volcanic islands located off the southeastern coast of Africa, east of Mozambique and northwest of Madagascar. The archipelago is divided between the sovereign state of the Union of Comoros composed of three islands named Grande Comore, Moheli, and Anjouan, and the French overseas department of Mayotte. The tropical climate of the Comoros islands is characterized by daytime temperatures around 26°C at sea level, with limited variation during the year, and by annual heavy rainfall (2,679 mm) with two seasons: a humid season from November to April, and a dry season from May to October.

**Figure 1:**
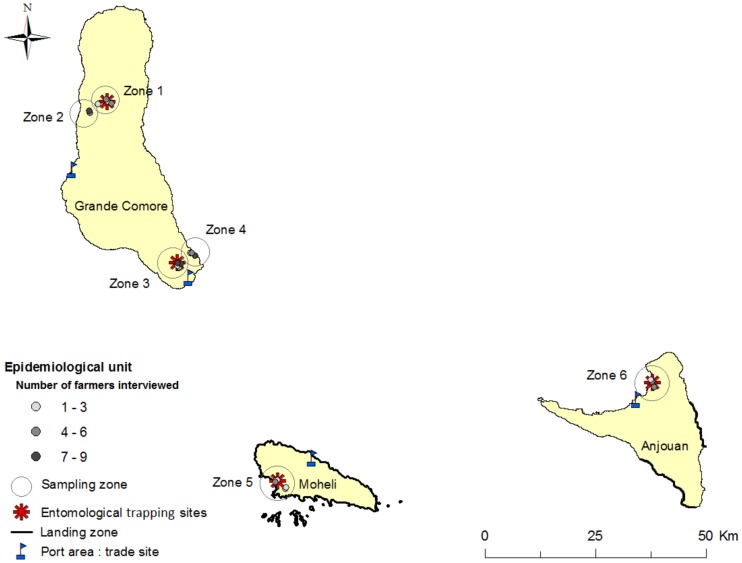
Location of the study zones. Location of the sampling sites (serological survey, entomological trapping and trade analysis). A landing zone is an area on the shore where boats drop passengers and animals off or pick them up.

Based on the results of a previous RVFV antibody prevalence study in 2009 [18], six zones were selected in the Union of Comoros (Figure 1). Four zones were selected on the island of Grande Comore: zones 1 and 2 located in the center of the island where low RVFV antibody prevalence was found, and zones 3 and 4 located in the south with high RVFV antibody prevalence [18]. Zones 2 and 4 are located along the coast (0–200 m above sea level (asl.)) where ruminants are mostly goats stall reared or ranging free within and outside villages. Zones 1 and 3 are located at a moderate altitude (500–650 m asl.) where ruminants are mostly cattle reared in pastures (zone 1) or raised in stalls in the forest (zone 3).

Zone 5, which was located on the southern coast of the island of Moheli, was selected because of its highest RVFV antibody prevalence observed during the 2009 survey [18]. On this island, cattle are reared in stalls on an old coconut plantation. Finally, in zone 6 located close to the airport on Anjouan island, cattle were raised in stalls in vegetable production areas.

### Animals and sampling

Five ml of whole blood was collected from the jugular vein of goats and cattle in Vacutainer tubes (Becton Dickinson, USA). Samples were allowed to clot at 15°C and serum was separated from whole blood by centrifugation; samples were stored in liquid nitrogen in the field and at −80°C in the laboratory.

### Livestock longitudinal survey

The livestock longitudinal survey was conducted in the six separate zones detailed in Figure 1. From 20 to 30 ruminants were individually identified in each zone using ear tags. The number of animals sampled per zone was based on the previous survey, with a RVFV antibody prevalence ranging from 20% to 50% [18] with 70% relative precision [23]. To avoid colostral immunity, animals were selected as follows: cattle were between 10 months and one-year of age, and small ruminants were between three to eight months of age. Animals were sampled monthly from April 2010 to August 2010 and every four months from August 2010 to August 2011. The first series of serological tests determined the RVF serological status of each sampled animal. Only RVFV antibody negative animals were included in the livestock longitudinal survey and continued to be sampled until their IgG RVFV antibody positive status and were then excluded from the study. When possible, new ruminants were included in the study to substitute lost, dead or RVFV IgG positive animals.

### Livestock cross-sectional survey

The RVFV antibody prevalence based on the different study zones was estimated in August 2011. The sample size was based on the previously estimated prevalence [18] with a relative precision of 20% and a confidence level of 95% giving a required minimum of 385 animals to be collected [23]. Without any particular Comorian livestock census, animals were selected on the farmer's willingness to cooperate during the study.

### Longitudinal entomological survey

Blood-sucking insects were sampled every four months from November 2010 to August 2011 along with the longitudinal serological survey using double-net goat baited traps placed from 4:00 pm to 10:00 am. The sampling was carried out for three consecutive days in the study zones numbered 1, 3, 5 and 6 (Figure 1). No sampling was performed in zones 2 and 4 for logistic reasons.

### Environmental data

In order to generate hypotheses on potential associations between the estimated RVFV incidence and prevalence with environmental risk factors for RVF infection, we collated climatic variables [24]. Two remotely-sensed MODerate-resolution Imaging Spectroradiometer (MODIS) data sets were sourced from the National Aeronautics and Space Administration (http://modis.gsfc.nasa.gov/), namely the Daytime Land Surface Temperature (DLST) and the Nighttime Land Surface Temperature (NLST), both with spatial and temporal resolution of 1 km and 8 days. In addition, rainfall data were obtained from the Malaria Early Warning System (MEWS) program, freely available in the MEWS repository (http://iridl.ldeo.columbia.edu/expert/SOURCES/.NOAA/.NCEP/.CPC/.FEWS/.Africa/.TEN-DAY/.RFEv2/.est_prcp/), with a spatial and temporal resolution of 11 km and 10 days respectively. DLST, NLST and rainfall values were extracted within a 5-km radius buffer around each farm corresponding to the maximum daily distance for cattle (grazing and watering). For each sampled animal that became RVFV antibody positive, MODIS and MEWS data recorded at the time of the seroconversion in the zone concerned were compared with MODIS and MEWS data recorded at the same time in the other zones.

### Trade survey

The aim of the trade survey was to estimate the movement of live animals between continental Africa and the Comoros archipelago and among the islands of the archipelago themselves. To date, only approximate figures are known without any quantitative data available [17]. The number of imported ruminants was collected monthly between November 2010 and August 2011 as follows i) the local veterinary authorities provided records of animal movements through the official ports of Moroni (Grande Comore), Fomboni (Moheli), and Mutsamudu (Anjouan), ii) one interviewer per island had the task of identifying undeclared animal arrivals on the coast, either in the field or from information provided by village chiefs.

### Laboratory tests

#### Detection of RVFV antibodies

Sera were first tested by two ELISAs (enzymelinked immunosorbent assay) the Immunoglobulin (Ig) G IDScreen RVF Competition ELISA (IdVet, France) and the IgM capture ELISA [25]. To confirm their status, each of the RVFV ELISA antibody positive samples and some randomly chosen RVFV ELISA negative samples were tested using the virus neutralizing test (VNT), considered as the gold standard method described by OIE [26]. Briefly, duplicates of two-fold serial dilutions of sera starting from 1∶5 were added to 100 TCID_50_ of Smithburn RVFV in 96-well microtiter plates and incubated for 1 h at 37°C. Next, 100,000 Vero cells were added to each well and the plates were incubated with 5% CO_2_ for 5–6 days at 37°C. Titers were expressed as the inverse highest dilutions giving 50% of CPE. A positive control serum was included. A serum sample with a titer of 1∶10 or higher was considered seropositive.

#### Morphological identification of insects

Specimens were collected by direct aspiration with a home-made vacuum system and anesthetized with chloroform. Each specimen was morphologically identified by microscopy in the field. Insects were pooled (1 to 10 individuals), per species per trap and per zone and stored in liquid nitrogen in the field and at −80°C in the laboratory. Engorged females were not included in the pool.

#### Detection of RVFV in insects

The pooled insects were ground up with 400 µl of PBS 1X (Phosphate Buffered Saline) twice for 30 seconds with two 3-mm diameter stainless beads using the TissueLyser system (Loudet, France) and transferred to a 96-well plate. Total RNA was extracted with the Biomek NX robot (Beckman Coulter, USA) using the NucleoSpin 96 Virus kit (Macherey-Nagel, Germany). For RVFV RNA detection, the L-Segment based SYBR-Green real time PCR was used [27], [28]. Ten-fold serial dilutions of a Smithburn strain which contained 10^8^ TCID_50_/ml were used as the standard curve for plate validation.

### Data analysis and statistics

All statistics were performed using R.3.0.1 [29]. For both Fisher's exact test and the Student-t test, a value of P<0.05 was considered significant.

A seroconversion was defined as an animal found with either a positive IgM ELISA result or a positive IgG ELISA result or both following a previous negative RVFV ELISA sample result.

#### Incidence rates and instantaneous risk of infection

The rate of instantaneous risk of infection (Txi) is described as:
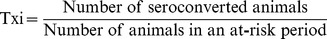



The incidence rate was determined using an Access database in the Laser format (available at http://livtools.cirad.fr/) [30]–[32] by calculating the instantaneous risk of infection (the risk that an animal will be infected in a given period) [30] taking into account the risk of seroconversion, death or lost animals. The number of animals in an at-risk period represents the total number of animals in a susceptible period of RVFV infection.

#### Incidence and seroprevalence analysis

To assess whether location had an effect on the RVFV antibody prevalence, different zones with specific ecosystems were included in the follow-up study. A Fisher's exact test was used to compare the difference in incidence of RVFV antibody acquisition and RVFV antibody prevalence between zones and islands. Incidence was analyzed and hypotheses were proposed about relationships with environmental and climate conditions.

#### Analysis of entomological data

A trapping session with no rain and/or wind was included in the data analysis even if no blood-sucking insects were collected. Student's t-Test was used for statistical analysis.

#### Trade survey

The movements of animals recorded between the three islands of the Union of Comoros were mapped, as well as their potential connection with continental Africa, Mayotte and Madagascar.

## Results

### Livestock longitudinal survey in relation with environmental data

A total of 191 ruminants (88 cattle and 103 goats) were included in the livestock longitudinal survey: 135 animals in Grande Comore, 27 in Moheli and 29 in Anjouan. Detection of RVFV antibodies (IgM and IgG) was performed by ELISA for a total of 849 serum samples over the duration of study.

Table 1 presents by date and per zone the number of animals that acquired RVFV antibodies over the duration of the livestock longitudinal survey. A total of 15 animals out of the 191 sampled acquired RVFV antibody during the study. Each of the 13 RVFV IgG ELISA positive samples were confirmed by VNT. Only one RVFV IgM ELISA positive sample was not confirmed by VNT (July 200, Moheli). This animal was confirmed RVFV IgG ELISA positive and VNT positive four months later in November 2010. Out of the 112 RVFV IgG ELISA negative samples randomly chosen, all were found negative by VNT. RVFV IgM antibodies acquisition was detected in three animals and RVFV IgG antibodies acquisition in 12 animals. Only one RVFV IgM ELISA positive animal in Moheli converted to RVFV IgG antibodies. The two others RVFV IgM ELISA positive ruminants were lost or slaughtered before the next sampling session (Table 1). Nine out of the 15, which acquired RVFV antibody, were recorded in Moheli, five in Grande Comore and one in Anjouan. Nine out of those fiftteen occurred during the dry season (six in Moheli, one in Anjouan, two in Grande Comore).

**Table 1:** Number of animals that acquired RVFV antibody per study zone during the livestock longitudinal survey, Union of Comoros, April 2010 to August 2011.

		Grande Comore																Moheli				Anjouan			
		Zone 1				Zone 2				Zone 3				Zone 4				Zone 5				Zone 6			
Sampling month		ELISA		n	VNT	ELISA		n	VNT	ELISA		n	VNT	ELISA		n	VNT	ELISA		n	VNT	ELISA		n	VNT
		IgM	IgG			IgM	IgG			IgM	IgG			IgM	IgG			IgM	IgG			IgM	IgG		
2010	April	-	-	15	-	-	-	25	-	-	-	23	-	-	-	25	-	-	-	26	0/1	-	-	23	-
	May	-	-	14	0/1	-	-	22	0/2	-	-	16	0/2	-	-	19	0/1	-	-	-	-	-	-	20	0/1
	June	**1** *	-	11	**1/1**	-	-	16	0/2	-	-	8	0/1	-	-	12	0/2	-	-	-	-	-	-	15	-
	July	-	-	9	-	-	-	13	0/2	**-**	**-**	-	-	-	-	-	-	**1**	**2**	16	**2/12**	**1** *	-	16	**1/6**
	November***	-	-	26	0/1	-	-	30	-	-	-	7	0/7	-	-	10	0/7	-	**3** **	9	3/10	-	-	19	0/2
2011	February	-	-	26	-	-	**1**	**29**	**1/9**	-	-	8	-	-	**2**	12	**2/7**	-	**1**	8	**1/1**	-	-	23	0/4
	May	-	-	16	0/2	-	-	20	-	-	**1**	13	**1/6**	-	-	8	0/5	-	-	7	0/5	-	-	21	0/4
	August	-	-	17	0/3	-	-	18	-	-	-	9	0/6	-	-	8	0/4	-	**3**	8	**3/8**	-	-	15	0/2

n: number of ruminants with RVFV antibodies negative status per sampling month, VNT: stands for Virus Neutralization Test,

*: animal slaughtered or lost,

**: included the RVFV IgM ELISA positive animal from July 2010,

***: new ruminants included in the study.

The overall annual incidence of RVFV antibody acquisition for the Union of Comoros was estimated at 17.54% (n[animal risk time] = 91), with a 95% confidence interval (CI) [8.95–26.14]) (Table 2). A significant difference was found when incidence of RVFV antibody acquisition was compared between zones (Fisher exact test, p<0.001) or between islands (Fisher exact test, p<0.001) (Table 2). Zone 5 (Moheli) incidence of RVFV antibody acquisition (72.3% 95% CI [0.255–1.000]) was significantly higher than in others zones (Table 2).

**Table 2:** Incidence of RVFV antibody acquisition per zone including statistical analysis, Union of Comoros 2011.

		Union of Comoros	Grande Comore (GC)					Moheli	Anjouan
Period			All GC zones	Zone 1	Zone 2	Zone 3	Zone 4	Zone 5	Zone 6
Annual	Incidence of antibody acquisition	0.175	0.082	0.051	0.055	0.071	0.227	0.723	0.058
	nrisk*	91	63	18	19	14	9	14	7
	95% CI	[0.089–0.261]	[0.010–0.155]	[0.000–0.163]	[0.000–00.152]	[0.000–0.212]	[0.000–0.542]	[0.255–1.000]	[0.000–0.173]
Rainy season (November–April)	Incidence of antibody acquisition	0.075	0.030	0.000	0.031	0.000	0.136	0.413	0.000
	nrisk*	92	64	28	32	23	15	10	20
	95% CI	[0.02–0.132]	[0.000–0.065]	[0.000–0.150]	[0.000–0.092]	[0.000–0.178]	[0.000–0.325]	[0.010–0.819]	[0.000–0.218]
Dry season (May–October)	Incidence of antibody acquisition	0.095	0.016	0.049	0.000	0.110	0.000	0.415	0.058
	nrisk*	93	62	21	25	9	7	14	17
	95% CI	[0.003–0.158]	[0.000–0.048]	[0.000–0.325]	[0.000–0.165]	[0.000–0.325]	[0.000–0.439]	[0.080–0.747]	[0.000–0.170]
Statistical analysis (using the dataset above)									
Comparison of dry season and rainy season incidence per zone [p-value]**		0.795	0.443	0.428	1	0.303	1	1	1
Comparison of the study zones incidence [p-value]**		Annual	<0.001						
		Rainy season	<0.001						
		Dry season	0.015						

CI: stands for Confidence Interval,

* nrisk represents the number of animals at risk in a susceptible RVFV infection period,

** Fisher exact test, p-value significant if p<0.05.

The incidence rate was determined using an Access database in the Laser format (available at http://livtools.cirad.fr/) [29]–[31] by calculating the instantaneous risk of infection (the risk that an animal will be infected in a given period) [29] taking into account the risk of seroconversion, death or lost animals. The number of animals in an at-risk period represents the total number of animals in a susceptible period of RVFV infection.

The statistical analysis did not reveal any significant difference in incidence of RVFV antibody acquisition between the rainy (from November to April,) and the dry season (from May to October) either for the Union of Comoros as a whole or per zone (Table 2). DLST, NLST (MODIS data) and cumulative rainfall (MEWS data) were similar in all six zones at the time of fourteen out of fifteen seroconversions occurred. There was one exception when the last RVFV seroconversion was recorded in Grande Comore in May 2011 (zone 3). Between March and May 2011, DLST, NLST and cumulative rainfall recorded in Grande Comore (29°C, 25°C and 730 mm respectively) were higher than those recorded in Moheli and Anjouan at the same time (DLST : 24°C, NLST : 22°C and cumulative rainfall : 420 mm). No RVFV seroconversion was recorded on Moheli and Anjouan during this period.

### Livestock cross-sectional study

In August 2011, to determine the RVFV antibody prevalence, a total of 275 ruminant samples (i.e. 163 cattle and 112 goats) were tested for the presence of RVF IgG antibodies. A total of 37 ruminants (20 cattle and 17 goats) came from the longitudinal follow-up study and 238 ruminants (143 cattle and 95 goats) were randomly selected in the six separate study zones.

The overall RVFV antibody prevalence in the Union of Comoros study zones in 2011 was 27.6% (n = 275, 95% CI, [22.3–32.9]). We found a significant difference of RVFV antibody prevalence between islands (Fisher exact test, p = 0.007), with a higher RVFV antibody prevalence in Moheli (45.8%, 95% CI, [33.7–57.9], Table 3).

**Table 3:** RVFV antibody prevalence in the Union of Comoros, 2011.

RVFV antibody prevalence			Statistical analysis [p-value]		
			Grande Comore	Moheli	Anjouan
Grande Comore	prevalence	0.247			
	n	174			
	95% CI	[0.183–0.311]			
Moheli	prevalence	0.458	0.008*		
	n	48			
	95% CI	[0.317–0.599]			
Anjouan	prevalence	0.207	0.683	0.013*	
	n	53			
	95% CI	[0.098–0.317]			
Union of Comoros	prevalence	0.276	0.007**		
	n	275			
	95% CI	[0.223–0.329]			

CI: stands for Confidence Interval,

*Fisher exact test, 2 by 2 comparison, p-value significant if p<0.05,

** Fisher exact test, multiple comparison, p-value significant if p<0.05.

### Insect trapping and RVFV detection

Twelve trapping days were conducted in each of the four zones under study (zones 1, 3, 5 and 6, see Figure 1). Blood-sucking insects were trapped in five out of the twelve trapping days in central Grande Comore (zone 1), in eight trapping days in southern Grande Comore (zone 3), eleven trapping days in Moheli (zone 5), and in seven trapping days in Anjouan (zone 6) (Table 4). Out of the 1,568 blood sucking insects caught with the double-net goat baited trap, 1,548 were identified as mosquitoes and 20 were identified as *Stomoxys niger*. A total of 1,133 insects were collected in Moheli (zone 5), 291 in Anjouan (zone 6), 108 in southern Grande Comore (zone 3) and 36 in central Grande Comore (zone 1). Although the number of comparisons was not large, the average number of trapped mosquitoes per trapping day per zone was significantly higher in Moheli (average was 113 insects) and Anjouan (average was 42 insects) compared to Grande Comore zones 1 (average was 7 insects) and zone 3 (average was 14 insects) (Table 5).

**Table 4:** Diversity and number of blood-sucking insects caught with a double baited net per trapping day and per zone, Union of Comoros, 2011.

		Grande Comore		Moheli	Anjouan	
Genus	species	Zone 1	Zone 3	Zone 5	Zone 6	Total number
*Stomoxis*	*niger*	0	0	11	9	20
*Aedes*	*aegypti*	1	4	8	15	28
*Aedes*	*cartroni*	0	0	504	48	552
*Aedes*	*circumluteolus*	0	0	0	10	10
*Aedes*	*simpsoni*	0	20	1	0	21
*Aedes*	*vittatus*	0	3	13	6	22
*Anopheles*	*arabiensis*	0	0	2	1	3
*Anopheles*	*coustani*	0	0	4	0	4
*Anopheles*	*sp*	0	0	25	0	25
*Culex*	*carleti*	0	1	9	0	10
*Culex*	*quinquefasciatus*	0	1	12	34	47
*Culex*	*sp*	0	0	12	0	12
*Eretmapodites*	*subsimplicipes/quinquevittatus*	35	77	524	168	804
*Uranotaenia*	*pandani*	0	2	6	0	8
*Mansonia*	*uniformis*	0	0	2	0	2
Total number of blood-sucking insects caught (effective trapping days*)		36(5)	108(8)	1133(11)	291(7)	1568(31)

*number of effective trapping days, i.e. days with the right climatic conditions (no wind or rain) to catch insects.

**Table 5:** Comparison of average number of mosquitoes caught per trapping day per zone (Student's t-Test), Union of Comoros, 2011.

		Grande Comore		Moheli
		Zone 1	Zone 3	Zone 5
Anjouan	Zone 6	p = 0.005*	p = 0.012*	p = 0.116
Moheli	Zone 5	p = 0.020*	p = 0.031*	-
Grande Comore	Zone 3	p = 0.280	-	-

* p-value significant if p<0.05.

The diversity and number of blood-sucking insects caught with the double net goat baited trap per trapping day per zone are presented in Table 4. A total of seven genera and 16 species were caught of which 14 could be morphologically identified. Fifteen species out of the 16 caught were collected in Moheli (zone 5), nine species were collected in Anjouan (zone 6), three and eight in central and southern Grande Comore respectively (zone 1 and zone 3). Eighty-seven percent of the total number of insects caught belonged to three species with 52% belonging to two *Eretmapodites* species (*E. quinquevittatus* and *E. subsimplicipes*,) and 35% to *Aedes cartroni*.

No RVFV RNA was detected in any of the 442 pools tested.

### Trade survey

**Figure 2:**
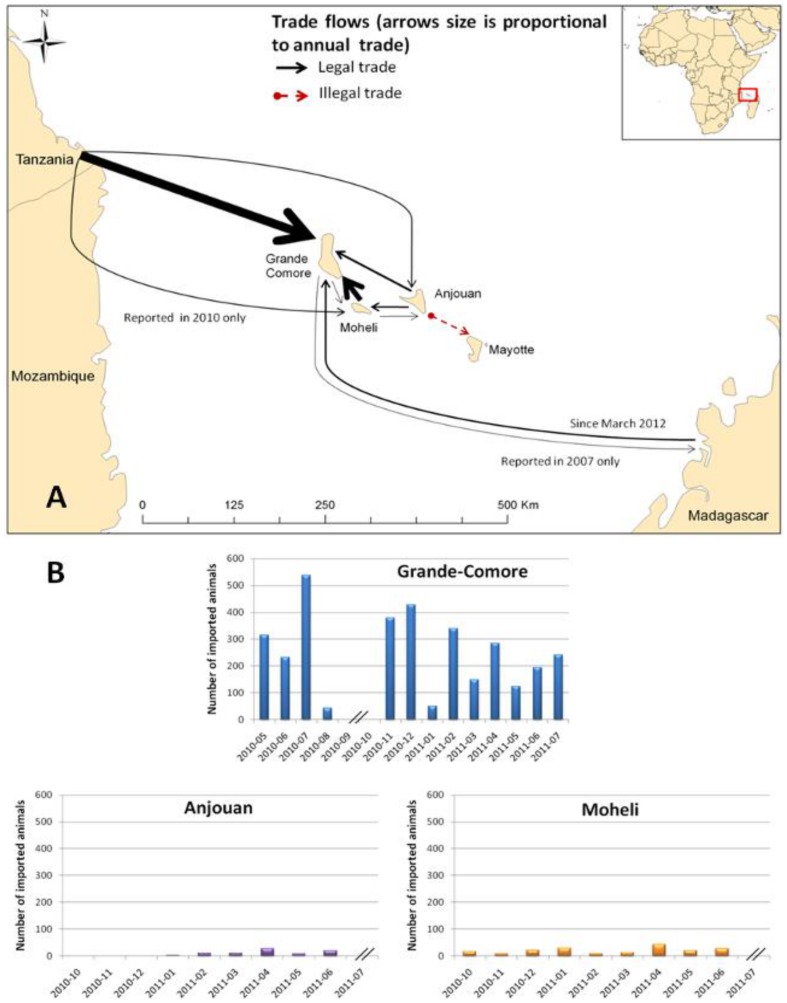
Trade in live animals between the Comoros archipelago, Madagascar and East Africa between 2007 and 2012. Trade in live animals between the Comoros archipelago, Madagascar and East Africa between 2007 and 2012 (Figure 2a) and imported animal dynamics (Figure 2b). Data used to create this map were gathered in surveys, or came from official sources or from personal communications.

The study highlighted movements of live ruminants between the three islands of the Union of Comoros, the African mainland, Mayotte and Madagascar (Figure 2). Data recorded by veterinarians and technicians showed movements of live ruminants from i) the east coast of Africa to Union of Comoros and ii) between the three islands of the Union of Comoros (Figure 2A.). Animals were observed being landed on beaches without any controls or in secondary “ports” like Chindini in the south of Grande Comore. Figure 2B represents the dynamics of live animal importations in Union of Comoros from May 2010 to July 2011. We recorded up to ten fold more ruminants imported in Grande Comore than in Moheli or Anjouan.

## Discussion

Rift Valley fever was detected for the first time in Grande Comore in the human population in 2007 [14] and in livestock in 2009 [18]. Our study demonstrates that RVFV is still circulating in the Union of Comoros despite of the absence of apparent clinical signs in livestock.

Fifteen RVFV seroconversions were observed in the Union of Comoros between 2010 and 2011 giving an overall incidence of RVFV antibody acquisition of 17.5%. These results suggest continuous circulation of RVFV on the three islands. However, significant differences in incidence were observed between islands (p<0.001). The incidence of RVFV antibody acquisition was higher in Moheli (72.3%) than in Anjouan (5.8%) and in Grande Comore (8.2%). This is in accordance with differences in RVFV antibody prevalence between the Union of Comoros islands recorded in 2009 and 2011. In 2011, RVFV antibody prevalence in Anjouan was still below the one in Grande Comore, whereas RVFV antibody prevalence remained the highest in Moheli. However, in Grande Comore and Anjouan RVFV antibody prevalence in 2011 appeared to have decreased whereas in Moheli, RVFV antibody prevalence remained similar to the level recorded in 2009, despite herd replacement estimated at 12% (L. Cavalerie, personal communication). These results suggest the existence of island specific RVF circulation patterns.

Seasonality of the incidence of RVFV antibody acquisition needs to be explored. The Comorian livestock farming characteristics (small herd size and small total number of ruminants) as well as the field issues did not allow a sufficient number of young ruminants (nrisk too small) reducing the power of the statistical analysis.

No clinical signs were reported in the Union of Comoros during the period of our study, as reported in Madagascar, Tanzania, and Mozambique in recent years [33]–[35], but the fifteen seroconversions observed suggest that RVFV could be circulating in the Comorian environment thanks to local mosquito-mammalian host cycles even if the numbers of caught mosquitoes were not large nor positive for RVF RNA. Out of the 1,568 blood-sucking insects caught, none were found to be RVFV RNA positive by PCR but in the absence of RVF outbreaks, chances of detecting RVFV in vector populations are known to be very low [36]. In 1978, Bruhnes described 30 mosquito species in the Union of Comoros [22]. Four of them: *Ae. aegypti*, *Ae. fowleri*, *Ae. circumluteolus* and *Cx. quinquefasciatus* are considered as RVFV potential vectors because the virus has been already isolated in these species in the field and because of their capacity to transmit RVFV under laboratory conditions [37]–[39]. All these species, except *Ae. fowleri*, have been caught at least on one island during our study, suggesting a role for this mosquito species to be involved in the transmission cycle on each of the islands. Five other mosquito species caught during our study, *Er. quinquevittatus*, *An. arabiensis*, *M. uniformis*, *An. coustani* and *Ae. simpsoni* were previously identified as RVFV RNA positive by PCR in the field [40]–[44]. *An. coustani* and *Ae. simpsoni* were found RVFV RNA carrier for the first time in the Indian Ocean area: respectively in Madagascar in 2011 and in Mayotte in 2009 [43], [44]. Thus, some of these mosquito species may play a role in RVFV transmission in the Union of Comoros. Geological inaccessibility, sampling design and climatic conditions likely explain the small number of specimens caught and the heterogeneity of entomological findings between islands [45]. These volcanic islands are characterized by a tropical climate with only slight variations in daily temperatures and abundant rainfalls, which theoretically should enable populations of Culicidae species to persist throughout the year. Nevertheless, each island has its own environmental characteristics, as the age of the three islands decreases westward: Moheli is 2.73±0.20 million years old, Anjouan, 1.18±0.03 million years old, and Grande Comore is 0.13±0.02 million years old [46].

On Moheli and Anjouan, the oldest islands, the landscape includes permanent rivers [47] and, as a result, many artificial and natural breeding mosquito sites exist. Moheli has a wide variety of natural and artificial sites in which mosquitoes can breed all year round [47], [48]. The presence of clay, resulting from the decomposition of volcanic soils, ensures the presence of abundant surface water impoundments. It allows the cultivation of irrigated rice hence and favors the development of diversified mosquito populations [47]. A greater number of mosquito species were caught in Moheli (15 species) than in the other islands which is in agreement with Brunhes' inventory in 1978, including two mosquito species known as RVFV potential vectors. Thus, favorable conditions for RVFV persistence being present a better chance for a possible RVFV cycle involving vectors and animals is suggested.

The abundance of mosquitoes trapped in Anjouan (zone 6) was similar to that in Moheli (zone 5) and three mosquito species known as RVFV potential vector have been caught during our study. However, RVFV antibody prevalence in Anjouan was the lowest and appeared to be decreasing. Moreover in 2011, only one ruminant exhibited a RVFV seroconversion. Anjouan shares some similar environmental characteristics with Moheli that could allow mosquitoes to survive all year round but Anjouan has some characteristics that could limit the circulation of RVFV. For example, the landscape is comprised of hill slopes and irrigated field rice is not cultivated on the island. Ruminants are mainly reared in stalls in the highlands in the eastern part of the island. For that reason, the probability of contact between infected vectors and ruminants may be lower in Anjouan than in Moheli and the maintenance of a vector-ruminant cycle may be harder to get. More investigations in other cattle-rearing areas are thus needed to conclude on RVF circulation in Anjouan.

Incidence of RVFV antibody acquisition and the RVFV antibody prevalence in Grande Comore are hard to explain based only on entomological parameters. Presence of steep slopes with decomposed and highly permeable soils characterize Grande Comore, the youngest island of the country [47]. Surface water is rare and only artificial containers (such as tanks and troughs) and some natural breeding sites (such as coconut shells and hollow trees) enable the development of Culicidae. Our results were in accordance with these observations as fewer blood-sucking insects were caught in Grande Comore when compared to Moheli and Anjouan. Thus, mosquito abundance in Grande Comore was likely correlated with the number of breeding sites that appeared after rainy episodes, as observed for the seroconversions we detected in Grande Comore following on from a major increase in cumulative rainfall. Two out of eight mosquito species caught during our study have been described as RVFV potential vectors. Consequently, environmental conditions for a local mosquito-mammalian host cycle could be met after important rainy episodes but a continuous circulation of RVFV in Grande Comore all year round is less likely to happen. However, regular introductions of the virus through the arrival of live animals from Tanzania [49], Anjouan, and Moheli may play a role in the persistence of RVFV in Grande Comore.

Analysis of trade in live animals confirmed observations reported by Cêtre et al., in 2012 in an overview of the movement of live ruminants between east Africa and the Comoros archipelago, as well as within the archipelago. Per year, more than 3000 live ruminants are imported from Tanzania (Chief Veterinary Officer of Comorian Vet services, personal communication), where RVF is endemic [34]. These animals enter the Union, mostly Grande Comore, without any quarantine or clinical examination. The risk of the introduction of new exotic strains of RVFV is consequently quite high and could affect the country in the same way as many other diseases in the past [19]. Tanzanian ruminants are imported for “great weddings” which are usually celebrated in July and August in Grande Comore. During these traditional weddings, villagers sacrifice ruminants without any particular sanitary rules. However, no major cases in humans and no ruminant seroconversions were reported during the “great weddings” period during our study but to date, human and veterinary health surveillance networks remain not very efficient. Occasional imports of Tanzanian ruminants into Moheli and Anjouan have also been reported; so new RVFV strains could have been also introduced on these islands. The regular introduction of live ruminants from Anjouan and Moheli could also contribute to the regular introduction of RVFV in Grande Comore as well.

Rift Valley fever epidemiology in the Union of Comoros is complex and further virological investigations should help to explain the origin of the RVFV strain(s) circulating within the islands. However, based on the results of the present study, RVFV seems hardly to persist on Grande Comore through a local vector cycle only but repeated reintroduction of viruses is possible. The situation regarding Rift Valley fever in Anjouan and Moheli appeared to look like that in Mayotte, Madagascar, Tanzania, and Mozambique [33]–[35], [50] where RVFV seroconversions have also been observed in the dry season without any apparent clinical signs. These findings could identify Moheli and Anjouan as endemic areas for RVFV. Given the incidence of RVFV seroconversions and antibody prevalence, RVFV is more likely to be circulating in Moheli than in Anjouan. However, additional data are needed to firmly conclude on the circulation of RVFV in the Union of Comoros. Wildlife such as bats and lemur species in our zone should be investigated even though no wildlife reservoir has been identified in any other country so far [51], [52].

Rift Valley fever is still a burden for the Union of Comoros as new human cases were diagnosed as RVFV positive in 2011 and in 2012 either by IgM or RVFV RNA detection with clinical signs [53], [54]. The real impact of the disease on human health and on the national economy is still unknown. Human and veterinary health networks need to be strengthened including the establishment of quarantine for imported ruminants.
